# Supplementary material for: Nationwide multi-centric prospective study for the identification of biomarkers to predict the treatment responses of nivolumab through comprehensive analyses of pretreatment plasma exosome mRNAs from head and neck cancer patients (BIONEXT study)
Source: Front Immunol. 2025 Jan 10;15:1464419. doi: 10.3389/fimmu.2024.1464419 (PMC11758179; doi:10.3389/fimmu.2024.1464419)
Supplement: Supplementary file 1 [file Table1.docx]

Supplementary Table S1. Primer sequences

| Species | Gene | Sense (5' to 3') | Antisense (5' to 3') |
| --- | --- | --- | --- |
| Human | ACTB | tttttcctggcacccagcacaat | tttttgccgatccacacggagtact |
| Human | CD3D | aagactggacctgggaaaacg | tcaacagagcttgtgtgtcg |
| Human | COL10A1 | tcccaatgccgagtcaaatg | acattggagccactaggaatcc |
| Human | CTSW | tcaccgtgaccatcaacatg | tgacttgacgctgccaaaac |
| Human | FAM76A | tcagtaccaggaatcgcagatg | tttggcctgcagttgttctg |
| Human | GAPDH | cccactcctccacctttgac | cataccaggaaatgagcttgacaa |
| Human | HLA-DQA1 | acatggctgtggcaaaacac | tgtgacctcaggaacctcattg |
| Human | HLA-DRA | tggacgatttgccagctttg | agttggagcgctttgtcatg |
| Human | HLA-E | tggttgctgctgtgatatgg | acaagctgtgagactcagacc |
| Human | LOC283788 | aaaacacagtgctgggcttg | atccactcgctgctttgtac |
| Human | MFSD8 | ctggactcaagaacaagctgtg | acgctcgccaatctttttgg |
| Human | MPIG6B | agtaaaggaggaagagcccaag | tccagatccgcatagagcag |
| Human | MSH2 | tgggtgttttgtgccatgtg | tttcagccatgaacgtggag |
| Human | RABL2B | aacctgagcacctggtatacag | tttattggccaccacgatgc |
| Human | RPL23AP7 | atgcagaagtcatgctggtg | tttggcttcggctttaggag |
| Human | SLC25A13 | tgccaaagcatgctttctgc | acctgcccatcttcatttgc |
| Human | TAF4B | aacagctggaactggtttgc | tggcttttcaggttgcagtg |
| Human | TCF7 | acctgaagacacaagcagagtc | acagcatgaaggcattgagg |
| Human | TESK2 | ttccccgcacagagaatttc | aaaagatgggcgcagtttgg |
| Human | TGIF1 | ttttggctcgtccatcagtg | tttttggccgctatctgctg |
| Human | TNFRSF13C | accccatcttttgctacagc | atgcccaaggtttgcatgtc |
| Human | ZNF480 | gtgagtattcaggcctttcagc | ttgtgtctgaatgccttgcc |
