# Supplementary material for: Nationwide multi-centric prospective study for the identification of biomarkers to predict the treatment responses of nivolumab through comprehensive analyses of pretreatment plasma exosome mRNAs from head and neck cancer patients (BIONEXT study)
Source: Front Immunol. 2025 Jan 10;15:1464419. doi: 10.3389/fimmu.2024.1464419 (PMC11758179; doi:10.3389/fimmu.2024.1464419)
Supplement: Supplementary file 2 [file Table2.docx]

Supplementary Table S2. Patient and tumor characteristics.

| Characteristics | *N* = 104 |
| --- | --- |
| Median age (range) , -years | 67.5 (35-82) |
| Sex -no.(%) |  |
| male | 92(88) |
| female | 12(12) |
| ECOG-performance status, -no.(%) |  |
| 0 | 68(65) |
| 1 | 31(30) |
| 2 | 5(5) |
| Extent of disease, -no.(%) |  |
| Locoregionally recurrence only | 60(58) |
| Distant metastasis | 44(42) |
| Primary cancer site, -no.(%) |  |
| Nasal and paranasal cavity | 9(9) |
| Oral cavity | 31(30) |
| Nasopharynx | 7(7) |
| Oropharynx (p16+) | 5(5) |
| Oropharynx (p16-) | 8(8) |
| Hypopharynx | 31(30) |
| Larynx (supraglottic) | 13(13) |
| Platinum agents previously administered, -no.(%) |  |
| CDDP | 86(83) |
| CBDCA | 21(20) |
| CDGP | 4(4) |
| No. of previous lines of systemic cancer therapy, -no.(%) |  |
| 1 | 73(70) |
| 2 | 27(26) |
| 3 | 4(4) |

CDDP, cisplatin; CBDCA, carboplatin; CDGP, nedaplatin
