# Supplementary material for: Nationwide multi-centric prospective study for the identification of biomarkers to predict the treatment responses of nivolumab through comprehensive analyses of pretreatment plasma exosome mRNAs from head and neck cancer patients (BIONEXT study)
Source: Front Immunol. 2025 Jan 10;15:1464419. doi: 10.3389/fimmu.2024.1464419 (PMC11758179; doi:10.3389/fimmu.2024.1464419)
Supplement: Supplementary file 3 [file Table3.docx]

Supplementary Table S3. Candidate best overall response predicting biomarkers

| Comparison | Gene | logFC | logCPM | F | *P*-value | FDR | AUC |
| --- | --- | --- | --- | --- | --- | --- | --- |
| PR vs. PD | TAF4B | -2.658 | 2.8695 | 13.179 | 0.001 | 0.992 | 0.9545 |
|  | TESK2 | -1.564 | 2.827 | 5.0272 | 0.032 | 0.992 | 0.9545 |
|  | MFSD8 | -1.729 | 3.0082 | 6.7906 | 0.0138 | 0.992 | 0.9545 |
|  | ZNF480 | -1.755 | 3.2126 | 9.8931 | 0.0036 | 0.992 | 0.9394 |
|  | FAM76A | -2.642 | 2.6952 | 11.335 | 0.002 | 0.992 | 0.9394 |
|  | TGIF1 | -1.718 | 3.2614 | 7.0577 | 0.0122 | 0.992 | 0.9318 |
|  | TNFRSF13C | -2.169 | 2.4797 | 6.4285 | 0.0163 | 0.992 | 0.9242 |
|  | LOC283788 | -2.761 | 2.8714 | 11.072 | 0.0022 | 0.992 | 0.9242 |
|  | SLC25A13 | -2.421 | 2.9492 | 9.1698 | 0.0048 | 0.992 | 0.9091 |
|  | HLA-DQA1 | 0.9238 | 3.5784 | 1.8142 | 0.1874 | 0.992 | 0.9015 |
|  | COL10A1 | -2.917 | 3.1403 | 10.181 | 0.0032 | 0.992 | 0.9015 |
|  | RPL23AP7 | 2.1722 | 3.7521 | 12.158 | 0.0014 | 0.992 | 0.8636 |
|  | CD3D | -1.244 | 2.9224 | 2.6487 | 0.1134 | 0.992 | 0.8485 |
|  | TCF7 | -1.224 | 4.5009 | 5.5421 | 0.0248 | 0.992 | 0.8409 |
|  | HLA-E | 3.5849 | 5.1057 | 9.1891 | 0.0048 | 0.992 | 0.8333 |
|  | HLA-DRA | -0.999 | 3.9583 | 2.3496 | 0.1351 | 0.992 | 0.7879 |
| PR vs. SD/PD | MSH2 | 0.9593 | 3.3172 | 4.0467 | 0.0526 | 0.9982 | 0.7424 |
| PR/SD vs. PD | RABL2B | -1.668 | 3.1032 | 9.4917 | 0.0042 | 0.9401 | 0.7424 |
|  | CTSW | 1.7366 | 5.465 | 15.124 | 0.0005 | 0.9401 | 0.7348 |
|  | MPIG6B | 1.9582 | 3.5563 | 9.3287 | 0.0045 | 0.9401 | 0.7121 |

PR, partial response; PD, progressive disease; SD, stable disease; FC, fold change; CPM, count per million; F, F-statistic values of quasi-likelihood F-test; FDR, false discovery rate, AUC, area under the ROC curve
