# Supplementary material for: Nationwide multi-centric prospective study for the identification of biomarkers to predict the treatment responses of nivolumab through comprehensive analyses of pretreatment plasma exosome mRNAs from head and neck cancer patients (BIONEXT study)
Source: Front Immunol. 2025 Jan 10;15:1464419. doi: 10.3389/fimmu.2024.1464419 (PMC11758179; doi:10.3389/fimmu.2024.1464419)
Supplement: Supplementary file 4 [file Table4.docx]

Supplementary Table S4. Candidate response-predicting biomarkers (assessed in responder vs. non-responder groups)

| Parameters | AUC | threshold | sensitivity | specificity | ppv | npv |
| --- | --- | --- | --- | --- | --- | --- |
| HLA-E | 0.729 (0.605-0.853) | -0.282 | 0.765 | 0.632 | 0.903 | 0.375 |
| ACTB | 0.673 (0.547-0.800) | 4.123 | 0.659 | 0.632 | 0.889 | 0.293 |
| MPIG6B | 0.672 (0.548-0.797) | -0.92 | 0.612 | 0.684 | 0.897 | 0.283 |
| RABL2B | 0.614 (0.452-0.777) | -7.296 | 0.624 | 0.632 | 0.883 | 0.273 |
| TNFRSF13C | 0.614 (0.480-0.748) | -9.185 | 0.624 | 0.579 | 0.869 | 0.256 |
| ZNF480 | 0.593 (0.451-0.734) | -8.132 | 0.753 | 0.526 | 0.877 | 0.323 |
| NLR | 0.591 (0.464-0.719) | 4.862 | 0.541 | 0.632 | 0.868 | 0.235 |

AUC, area under the ROC curve; ppv, positive predictive value; npv, negative predictive value
